# Supplementary material for: Temporal and regulatory dynamics of the inner ear transcriptome during development in mice
Source: Sci Rep. 2022 Dec 7;12:21196. doi: 10.1038/s41598-022-25808-9 (PMC9729293; doi:10.1038/s41598-022-25808-9)
Supplement: Supplementary file 13 — Supplementary Figure S1. [file 41598_2022_25808_MOESM13_ESM.pdf]

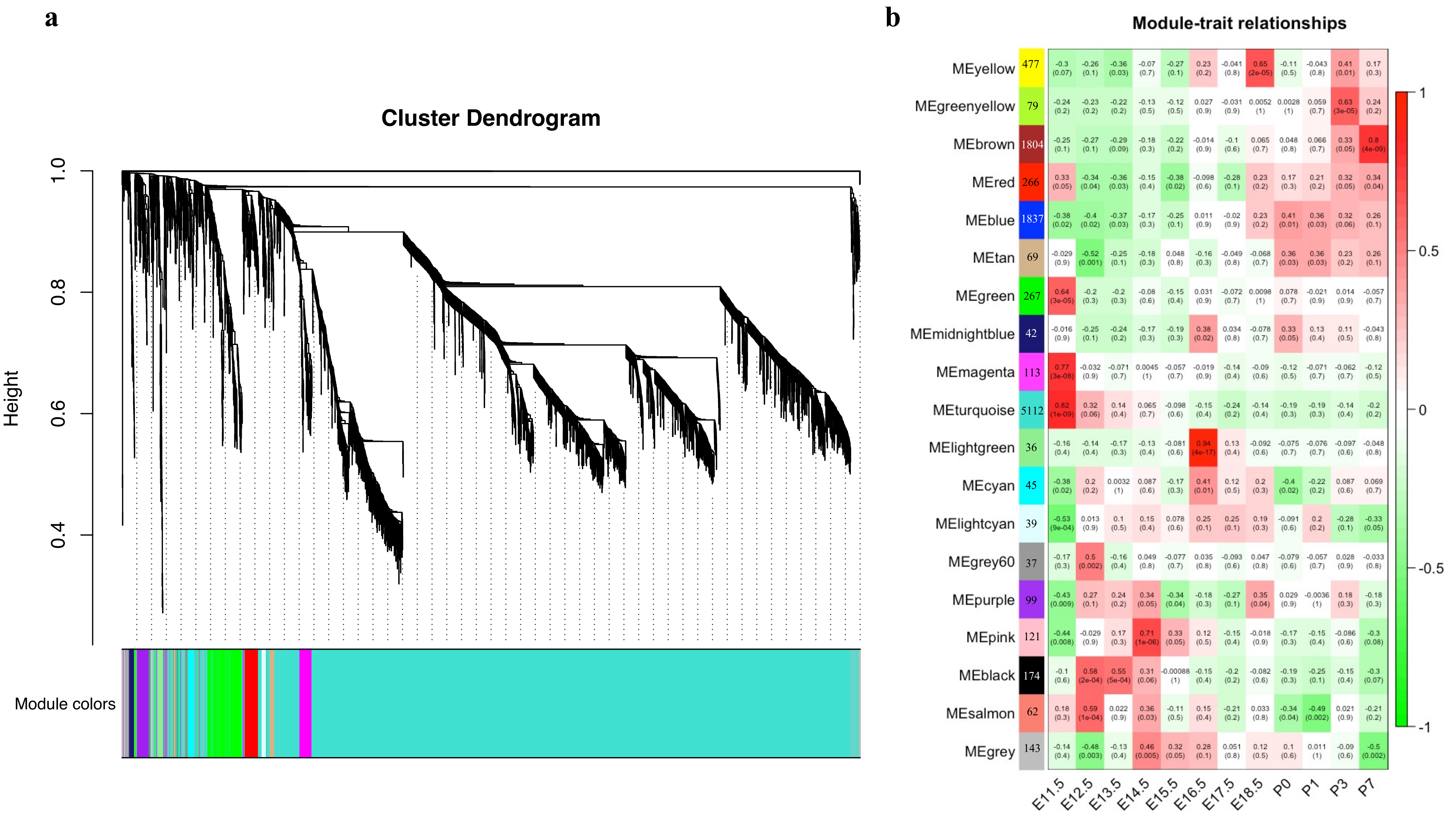

Figure S1. Co-expression network based on 10822 differentially expressed genes. (A) Hierarchical clustering of co-expression data. (B) Table of module–trait relationships. The value at the top of each square represents the correlation coefficient between the module eigengene and the trait with the correlation P-value in parentheses. The right panel is a color scale for module trait correlation from − 1 to 1.
